# MS-based lipidomics of human blood plasma: a community-initiated position paper to develop accepted guidelines^1^

**DOI:** 10.1194/jlr.S087163

**Published:** 2018-08-16

**Authors:** Bo Burla, Makoto Arita, Masanori Arita, Anne K. Bendt, Amaury Cazenave-Gassiot, Edward A. Dennis, Kim Ekroos, Xianlin Han, Kazutaka Ikeda, Gerhard Liebisch, Michelle K. Lin, Tze Ping Loh, Peter J. Meikle, Matej Orešič, Oswald Quehenberger, Andrej Shevchenko, Federico Torta, Michael J. O. Wakelam, Craig E. Wheelock, Markus R. Wenk

**Affiliations:** Singapore Lipidomics Incubator (SLING), Life Sciences Institute,* National University of Singapore, Singapore; Department of Biochemistry, YLL School of Medicine,|| National University of Singapore, Singapore; Laboratory for Metabolomics,† RIKEN Center for Integrative Medical Sciences, Yokohama, Japan; Cellular and Molecular Epigenetics Laboratory,†† Graduate School of Medical Life Science, Yokohama City University, Yokohama, Japan; Division of Physiological Chemistry and Metabolism,§ Keio University Faculty of Pharmacy, Tokyo, Japan; National Institute of Genetics,‡ Shizuoka, Japan and RIKEN Center for Sustainable Resource Science, Yokohama, Japan; Departments of Pharmacology and Chemistry and Biochemistry,# School of Medicine, University of California at San Diego, La Jolla, CA; Lipidomics Consulting Ltd.,$ Esbo, Finland; Barshop Institute for Longevity and Aging Studies and Department of Medicine-Diabetes,** University of Texas Health Science Center at San Antonio, San Antonio, TX; Institute of Clinical Chemistry and Laboratory Medicine,§§ University of Regensburg, Regensburg, Germany; Department of Laboratory Medicine,‡‡ National University Hospital, Singapore; Baker Heart and Diabetes Institute,|||| Melbourne, Victoria, Australia; Turku Centre for Biotechnology,## University of Turku and Åbo Akademi University, Turku, Finland and School of Medical Sciences, Örebro University, Örebro, Sweden; Departments of Pharmacology and Medicine,$$ School of Medicine, University of California at San Diego, La Jolla, CA; Max Planck Institute of Molecular Cell Biology and Genetics,*** Dresden, Germany; Babraham Institute,††† Cambridge, United Kingdom; Division of Physiological Chemistry 2,§§§ Department of Medical Biochemistry and Biophysics, Karolinska Institute, Stockholm, Sweden

**Keywords:** clinical trials, diagnostic tools, lipids, mass spectrometry, absolute concentrations, clinical research, data sharing, National Institute of Standards and Technology Standard Reference Material 1950, quality control

## Abstract

Human blood is a self-regenerating lipid-rich biological fluid that is routinely collected in hospital settings. The inventory of lipid molecules found in blood plasma (plasma lipidome) offers insights into individual metabolism and physiology in health and disease. Disturbances in the plasma lipidome also occur in conditions that are not directly linked to lipid metabolism; therefore, plasma lipidomics based on MS is an emerging tool in an array of clinical diagnostics and disease management. However, challenges exist in the translation of such lipidomic data to clinical applications. These relate to the reproducibility, accuracy, and precision of lipid quantitation, study design, sample handling, and data sharing. This position paper emerged from a workshop that initiated a community-led process to elaborate and define a set of generally accepted guidelines for quantitative MS-based lipidomics of blood plasma or serum, with harmonization of data acquired on different instrumentation platforms across independent laboratories as an ultimate goal. We hope that other fields may benefit from and follow such a precedent.

Blood plasma is a self-regenerating well-defined biological fluid that can be easily collected with minimal health risk. It is also rich in lipids and related metabolites, and its composition reflects diverse aspects of both metabolism and general human physiology in health and disease. Advances in MS, data processing algorithms and tools, databases, knowledge about lipid diversity, and the availability of a broad palette of high-quality synthetic standards have stimulated efforts toward the systematic quantification of plasma lipids in various clinical contexts. Such advances have also enabled the practical use of large biobanks assembled by generations of clinicians and clinical chemists to correlate lipid composition with the onset and progression of disease. In turn, this has triggered massive efforts toward the discovery of clinically relevant biomarkers (1–11). Although these efforts have produced some promising markers and means of monitoring the severity of disease, the fundamental conclusion was that, despite the diversity of pathophysiological disturbances, the plasma lipidome remains a tightly regulated and precisely defined constellation of lipid molecules. Thus, as for common clinical plasma indexes, the time has come to establish reference concentrations for individual lipids. Studies spearheaded by the LIPID MAPS consortium (12) and, more recently, by the National Institute of Standards and Technology (NIST) study group (13) have determined consensus values of plasma lipid concentrations in the NIST Standard Reference Material (SRM) 1950 plasma (**Fig. 1**). Efforts are underway to establish reference values for the concentration of various lipid species for individuals of different gender and ethnicity (14–20).

**Fig. 1.**
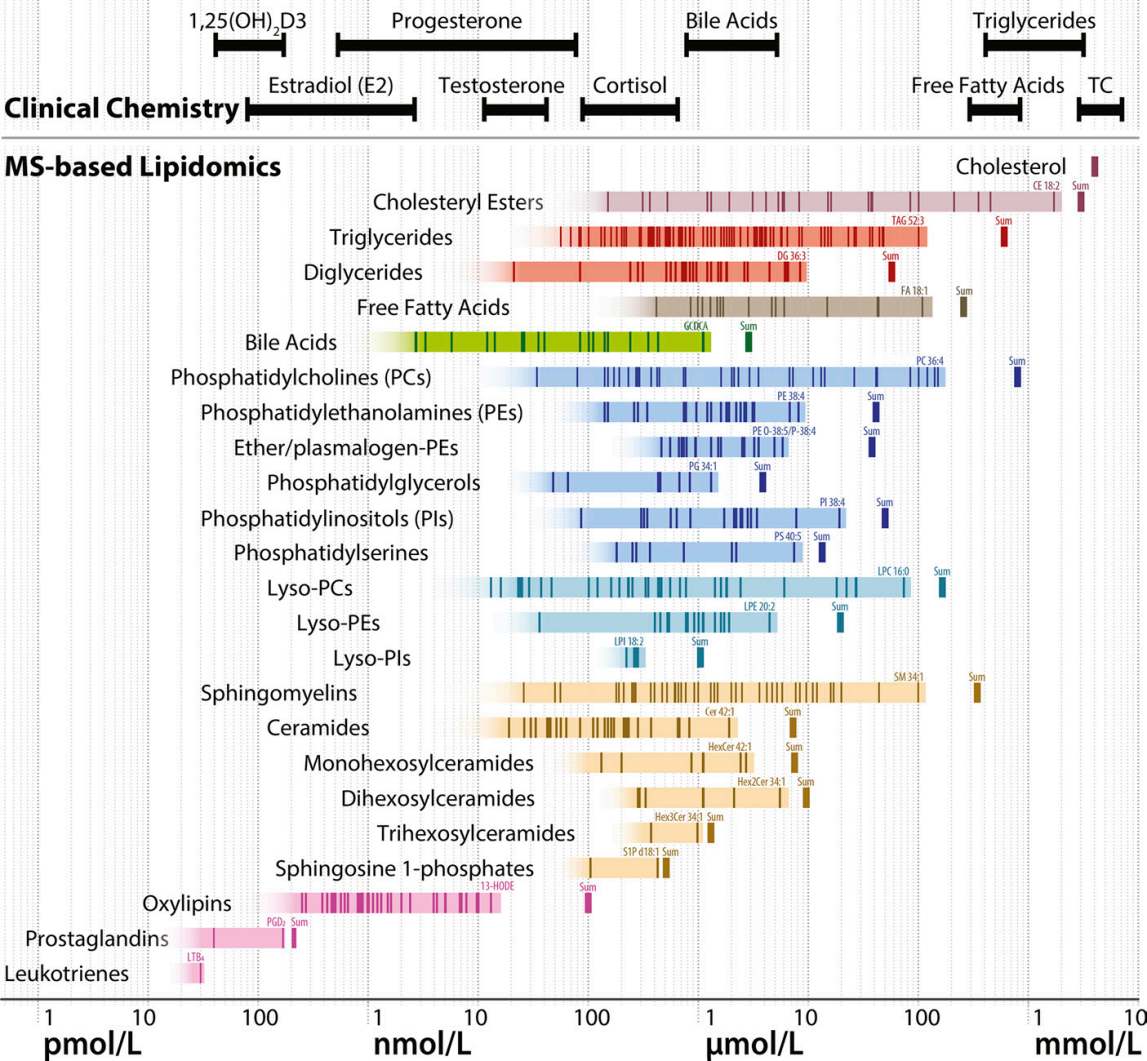
Concentrations of lipid species reported for the NIST SRM 1950 reference plasma. Each lipid species reported by at least three laboratories from the NIST SRM 1950 comparative plasma study (13) is indicated at its consensus concentration (median of means) as vertical dark lines within the shaded bars. Many of the shown species represent sums of species that can be further divided into more structurally resolved species, if analyzed with methods revealing higher structural information. The shaded boxes indicate the concentration range of the identified lipid species, illustrating that the actual number of lipid species and their concentration range are likely to be much larger in reality. The name of the highest abundant lipid species is displayed for each lipid class. The sum of the concentrations of individual lipid species of the lipid classes are indicated as vertical thick lines to the right of the shaded bars. Lipids measured in clinical chemistry laboratories are indicated at the top of the figure, with plasma or serum concentration ranges corresponding to clinical laboratory reference values in healthy adult people (139), except for triglycerides and total cholesterol (TC) where the 5th and 95th percentiles of the plasma concentrations in a large Dutch cohort are indicated (140). 1,25(OH)2D3, bioactive 1,25-dihydroxyvitamin D_3_.

We therefore speculate that the “starting phase” of plasma lipidomics is over. The lipidomics community should now make an effort to deliver concordant concentrations of individual lipids together with broad lipid class coverage, as these analyses are now routinely performed in dozens of laboratories worldwide.

Despite the overall success to date, the field faces several challenges (21). First, it is difficult to harmonize the published data and make them amenable to independent multidimensional data-mining by interested researchers. It appears that current efforts are filling selected pathophysiological niches, but hardly contribute to the understanding of compositional trends at a systemic level. Second, the quality of lipidomics data and the robustness of methodologies suffice for discovery research, but fall short of the common requirements for potential diagnostic applications (22). Communication between research and clinical communities remains to be fully developed and there is no system in place to assess and cross-correlate plasma lipidomic profiles obtained by different laboratories in various clinical settings. This leads to an odd (and strategically unacceptable) situation where a rapid increase in the total volume of produced data does not contribute to data refinement (23).

This position paper emerges from a workshop held in Singapore in April 2017 on this topic and whose participants committed themselves to communicating their workflows and generally agreed conclusions. The motivation to do so is founded on the belief that the community involved with MS-based lipid analysis should come together to set guidelines generally accepted in the field. To facilitate this process (possibly in an order of priority for applications), it was decided to strictly limit the discussion in this work to the lipidomic analysis of human blood (in particular, blood plasma and/or serum) and to MS as the main measurement technique, rather than other techniques, such as NMR. If successful, other applications would be expected to benefit and follow from such a precedent.

Different layers of quality assurance (QA) and quality control (QC) measures are prerequisites to obtain reproducible and quantitatively concordant datasets. Batch-to-batch variations are an inherent characteristic of high-throughput analytics, irrespective of the precise nature of the analysis. This is largely recognized in clinical diagnostics, where performance verification and QC measures, including external QA programs and proficiency testing, are put in place to detect significant deviations. In fact, clinical laboratories are mostly concerned about “between-methods bias”. Data are rarely merged among different laboratory methods unless well-harmonized, and we see no conceptual reason why data concordance could not be reached for the plasma lipidome. Different QA and QC methods have been developed for MS-based metabolomics and lipidomics (5, 24–27). However, the implementation of QA/QC strategies varies in both fields (23, 28, 29). Therefore, a community-initiated approach toward generally accepted guidelines for clinical application of plasma lipidomics seems pertinent, with an ultimate goal for harmonizing data acquired on different instrumentation platforms in independent laboratories. We appreciate the challenges involved in achieving this goal. This work mostly considers analyzing the core components of the plasma lipidome, and we understand that for some physiologically important, yet low abundant or unstable, lipids for which no reliable internal standards (ISTDs) or alternative analytical methods are available, this may not be feasible, as is the case for oxidized lipids (30–33).

Here, we propose that such laboratory practices could be adopted by a community largely representing research and development in the area of life sciences and also in clinical testing. Therefore, recommendations for potential future adoption are organized into three main categories: pre-analytics, analytics, and post-analytics. This short write-up is not intended to be comprehensive, particularly with respect to the various subcategories addressed here (**Fig. 2**). Instead, as introduced above, it should serve as a working document for a growing number of subscribers.

**Fig. 2.**
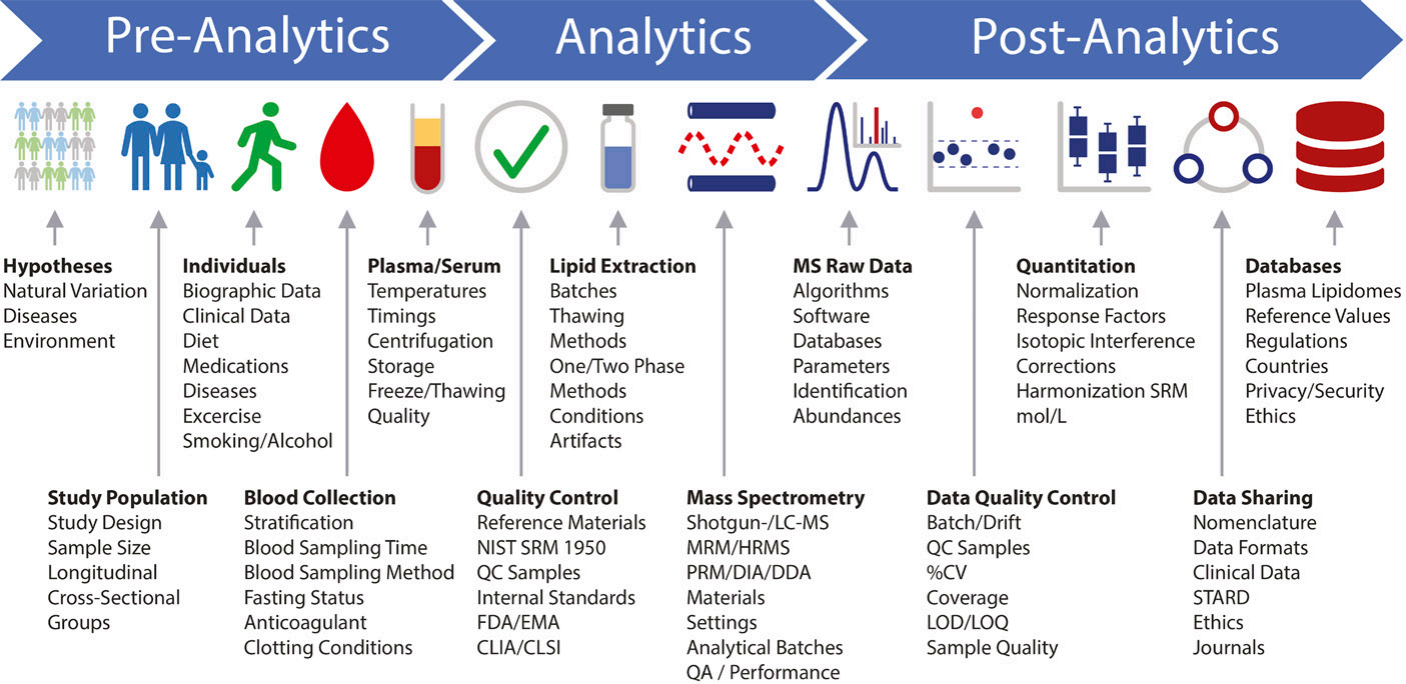
Human plasma lipidomics workflow. The major steps covered in this position paper are indicated together with important biographic parameters, sample preprocessing and analytical aspects, QC measures, and guidelines that should be considered and/or reported with quantitative plasma lipidomics datasets. HRMS, high-resolution MS.

## PRE-ANALYTICS

We define pre-analytics as “all procedures before the actual lipidomic analysis”. This includes study design, specification of the nature and origin of samples, collecting and communicating demographic and clinical data, and how plasma and serum are sampled and stored.

### Standards and guidelines

Relevant guidelines for bioanalytical method validation and performance include the Food and Drug Administration (FDA) Bioanalytical Method Validation Guidelines (34, 35), the European Medical Agency (EMA) Bioanalytical Method Validation Guideline on bioanalytical method validation (36), and the Japanese Ministry of Health, Labour, and Welfare (MHLW) Guideline on Bioanalytical Method Validation in Pharmaceutical Development (37). These guidelines were initially tailored for pharmaceutical, pharmacokinetic, and toxicokinetic applications and thus include MS as a methodology. However, many of the criteria and strategies mentioned in these guidelines are also applicable and relevant for the development and validation of lipidomic assays to be used in clinical research (38, 39). Following such guidelines will facilitate the development of clinical applications for plasma lipidomics.

Laboratory methods that are developed in-house are considered “laboratory-developed tests”. They need to undergo stringent validation processes as prescribed by certain standards, e.g., the International Standards Organization 15189 (40) and Clinical Laboratory Improvement Amendments (CLIA) (41), and subscribe to external QA programs for monitoring of their ongoing performance. These are required as part of accreditation of a routine clinical laboratory by the relevant regulatory authority. The same validation process and subscription to external QA programs are required each time a method is applied in a different laboratory.

Looking forward, guidelines and protocols used in clinical diagnostics and clinical chemistry will also be relevant for plasma lipidomic assays, including the International Standards Organization 15189 and CLIA laboratory protocols. These guidelines cover an extensive range of required topics for the accreditation of diagnostics and assays, and include training, QA/QC, administrative processes, infrastructure/facility design and management, human resources, auditing, and system design.

However, all of these clinical diagnostic guidelines do not, or only superficially, cover MS-based metabolomics and lipidomics. Furthermore, only a few MS-based methods have been published that were validated according to such guidelines (42–47). Recently, the Clinical Laboratory Standards Institute (CLSI) issued the CLSI C62-A guideline on MS in the clinical laboratory (48, 49). Considering such guidelines during assay development may improve the quality and adoptability of lipidomic assays for plasma analysis in clinical settings.

In this position paper, we aim to highlight the critical aspects of quantitative lipidomics of human plasma. The primary focus is the application of plasma lipidomics in high-quality clinical research and the identification of biomarkers. The use of plasma lipidomics in clinical diagnostics is a logical extension to that, but is currently still a rather distant scenario. Regardless of the application, current research and development into nucleic acid, protein, and metabolite biomarkers is likely changing clinical research and diagnostic procedures over time and thus will also require new or specific guidelines. Acceptance of new procedures and the willingness to define new guidelines will depend critically on the clinical performance, usefulness, simplicity, and applicability of novel methodologies, as well as on proper communication and documentation. It is therefore up to the respective communities to define standards in line with evolving practice in contemporary and future clinical research and development.

### Collection of demographics and clinical data

The value of a public plasma lipidomic database (e.g., for meta-analyses and the establishment of reference values) is highly dependent on the quality of the data associated with the samples. We encourage the community to put effort into collecting associated biographic and clinical data and to actively participate in the planning and implementation of novel regulations concerning data collection, anonymization, de-identification, and reporting (see also section Data sharing). Collected personal and clinical data intended for future use in publications (together with lipidomic data) should be defined at the time of application for approval by institutional review boards, so that the participants’ consent forms state that the information is being collected ethically, thereby allowing full use of the collected data.

We suggest that the minimum set of personal and clinical data collected along with plasma/serum samples should be subject age, gender, BMI, ethnicity, fasting status, and prescription medications, including drugs directly affecting lipid metabolism (e.g., nonsteroidal anti-inflammatory drugs, anticoagulants, and statins) and also drugs with insufficiently characterized metabolic impact (i.e., hormones, including contraceptives, steroids, and diuretics) (17, 50–52). It should also include significant medical conditions (e.g., affected with chronic disease). Recent research suggests that the spectrum of drugs affecting the lipidome composition is broad, and metabolic side-effects, being harmless per se, might bias the outcome of epidemiological studies. We therefore suggest providing detailed data on all given, prescribed, self-medicated drugs and health supplements together with lipidomics datasets and, if applicable, to analyze datasets for potential confounding effects of these medications. Specific populations, such as pediatric cohorts, may require different/additional sets of relevant variables.

The submission of additional parameters is strongly encouraged and for adults should include: diabetic/insulin status, HDL/LDL/triacylglycerol (TG) values, blood pressure, full blood count, C-peptide, C-reactive protein, smoking status, alcohol consumption, diet, intake of dietary supplements, type and frequency of exercise, and other information on lifestyle. Recoding of socio-economic indicators can also be of value, as these may offer information about dietary and environmental exposure. This data collection must be within the practice guidelines of local institutional review boards and legislation related to human biomedical research and personal data protection, but also with an outlook toward depositing the data in internationally accessible repositories. The latter generally mandates strict separation of identification keys from the individuals involved in the research.

### Plasma versus serum

Plasma and serum are two distinct matrices, and the lipid profiles of plasma and serum obtained from the same blood draw differ (53, 54). Serum is obtained from coagulated blood, whereby various compounds, including lipids and lipid-modifying enzymes, are released in extracellular vesicles or in soluble forms from platelets, leukocytes, and erythrocytes during the clotting process. The coagulation process therefore leads to generation or degradation of species in a lipid class-dependent manner. This can strongly affect the abundances of lysophospholipids (lyso-PLs), sphingosine 1-phosphates (S1Ps), prostaglandins, leukotrienes, resolvins, and other oxylipins, as opposed to major lipoprotein-bound TGs and cholesteryl esters (54–57). In clinical practice, serum is more widely used and may therefore be more suitable or acceptable for diagnostic applications. The measurement of lipids generated by the clotting process is also used to assess the treatment efficacy of anti-platelet drugs (58–60). However, plasma obtained from freshly drawn anticoagulated whole blood can be considered as the closest matrix to blood plasma in vivo. The use of capillary blood has several advantages for specific clinical applications, such as point of care and screening, allowing blood sampling without trained personnel. However, capillary blood, when collected via finger prick, is often contaminated with skin tissue fluid, applied cosmetics, and antiseptics, among others, and is prone to hemolysis. Therefore, we recommend the use of plasma obtained from venous blood for future lipidomic projects for better robustness and for comparing and interpreting physiological conditions.

### Blood collection and plasma/serum preparation

The method and timing of blood collection and plasma/serum preparation can have significant impact on downstream analyses (61, 62). Often neglected in clinical research, blood collection and plasma/serum preparation should be kept consistent between experimental groups, sites, and studies, and be reported. Because lipid concentrations can exhibit substantial circadian variations, the time point of blood collection should be kept consistent within a study (63). Venous blood should be collected using established protocols, with practices that minimize artifacts, such as hemolysis, clotting, platelet activation, and hemoconcentration from venous stasis (64, 65). Blood should preferably not be taken from infusion catheters (e.g., to avoid hemolysis and dilution of the blood by residual infusion solution). However, where unavoidable, discarding the initial volume of drawn blood may reduce artifacts (64).

Plasma should be prepared from whole blood collected directly in tubes containing dried or liquid anticoagulant to minimize clotting. Containers spray-coated with K_2_EDTA are routinely used in clinical practice; it is currently the most commonly used anticoagulant in lipidomic research. Other common anticoagulants include lithium heparin and citrate. The anticoagulant can have an impact on lipid extraction and ionization in MS, and blood collection tubes have been identified as a source of interference in metabolomics studies (66–69). Limited and partially contradicting data are available as to the effects of different anticoagulants on the lipidomic readout (56, 66, 70–72). The mechanism of the different anticoagulants, i.e., the calcium-chelating effects of EDTA and citrate, as opposed to heparin, may also be relevant to inhibit the calcium-dependent ex vivo formation or degradation of some lipid classes (56, 73). Of note, the NIST SRM 1950 plasma (discussed in detail later in this text) was prepared from lithium heparin-anticoagulated blood (74) and might not be a suitable reference for certain lipid classes that are produced or degraded in a calcium-dependent manner. Although more data on the effects of the anticoagulants are needed, it is important that the same anticoagulant is used throughout a study and among studies that will be compared, and to report the anticoagulant/collection tubes in detail. When using collection tubes with an anticoagulant solution (e.g., citrate), the tubes should be filled to the indicated volume to ensure the appropriate ratio of sample to anticoagulant and to avoid nonuniform dilution of the collected blood; this is a potential source of error and variation (71).

Collected anticoagulated whole blood should be processed consistently and as soon as possible to limit ex vivo metabolic processes that can affect the lipid profiles. Chilling of whole blood after collection is also generally advisable, especially when immediate processing after collection is not possible, as this substantially reduces ex vivo formation or degradation of certain lipid classes, e.g., S1P, lyso-PL, and eicosanoids (68, 72, 75–78). However, ex vivo cold exposure might lead to platelet activation and consequent release of platelet-derived lipid species into the plasma. On the other hand, plasma obtained from cooled whole blood has been reported to contain lower levels of specific platelet-derived eicosanoids (77). The authors hypothesized that cold-induced ex vivo platelet aggregation led to a more efficient removal of platelets from plasma during centrifugation and lowered the levels of these specific lipid species. Cooling could also have slowed down the enzymatic production and release of these lipids. Platelets are rich in lipids of diverse classes and, upon activation, produce and release various bioactive lipid species (79). Residual platelets in plasma can therefore confound the measured plasma levels of specific lipid species. High variability of residual platelet content can result from differences in plasma density/viscosity, which is reflected by the erythrocyte sedimentation rate, a parameter that varies between individuals and is increased in many inflammatory conditions (80, 81). The centrifugation conditions of whole blood also affect the number of residual platelets in plasma and the measured plasma metabolome (76, 82, 83). To ensure effective and consistent platelet removal, we therefore recommend using higher relative centrifugation forces than those sometimes used or suggested to prepare plasma, for example, 2,000 *g* for 15 min (54, 76, 82). However, we note that centrifugation also leads to platelet activation, which may be due to compaction as well as acceleration and deceleration forces (83). For serum preparation, the coagulation status of the collected blood, the clotting time and temperature, and the presence and type of clot activators in the collection tubes are important considerations, as they each can affect the lipid profiles of the generated serum (54).

Hemolysis can have significant effects on the plasma levels of certain lipid species, such as S1P and lyso-PL (68, 77, 84). Conditions causing increased erythrocyte fragility, such as specific erythrocyte membrane disorders (e.g., spherocytosis) and potentially also other disorders (e.g., type 2 diabetes), could cause increased hemolysis during plasma preparation (85, 86). Plasma preparation protocols will therefore have to be adjusted when studying conditions and lipids that may impact or be affected by hemolysis, respectively. Lipemia is defined as the presence of high levels of suspended lipoprotein particles resulting in blood sample turbidity. Lipemia can also cause analytical artifacts due to volume displacement by the particles and nonhomogeneity of the samples (87, 88).

Collectively, detailed information on sample collection and preparation conditions, as well as visible signs of sample quality (i.e., hemolysis and lipemia), should be reported for later data interpretation. Furthermore, validation of methods for robustness to such preanalytical variabilities will be helpful in defining practical sample collection protocols and in interpreting generated lipidomic data.

For certain plasma lipid species (i.e., oxylipins), it may be difficult to fully prevent sampling artifacts (e.g., partial platelet activation), and thus the interpretation of such data must consider possible biases and variabilities resulting from such preanalytical effects. In such cases, one could also consider using alternative or indirect approaches to assess the plasma levels of specific lipid species. For example, F2-isoprostanes, in particular 8-iso prostaglandin F_2α_, are highly-characterized prostaglandin-like compounds and are currently the best-studied markers of oxidative stress. These are routinely tested in urine as opposed to plasma (89).

These and other parameters may be of relevance for inter-site comparisons and data integration. Measures, such as using standard operating procedures, should be applied to limit potential variations during sampling, and clinical personnel should be encouraged to document and report any deviations from established procedures. If such controlled reporting is not feasible or realistic, for instance, in clinical settings, additional care is needed to avoid sampling bias, particularly across different experimental groups of which participants may be sampled under different settings. Statistical analysis should account for such possible variations in sampling procedures.

### Sample storage

Sample storage and the freezing chain during transport are crucial aspects of plasma metabolite profiling. Many metabolites are not stable in plasma and serum, especially at temperatures above −20°C (56, 68, 77, 90–92). A study from Haid et al. (93) found that the concentrations of specific lipids change during storage, even at −80°C, a widely used storage temperature for biological specimens. They explained it by possible nonenzymatic hydrolysis and oxidation of lipids (93). However, more data are needed to understand the limitations and possible measures to stabilize samples during long-term storage. For now, samples should be stored at −80°C or, if possible, at even lower temperatures. The containers used for sample storage should be airtight to prevent sublimation, which can also affect sample concentrations (94). Freeze-thaw cycles also affect specific lipid classes and should be kept at a minimum and constant within a study (19, 56, 92), unless contrary evidence is available for the stability of the measured analytes. Preparing aliquots directly after plasma/serum isolation will help to limit freeze-thaw cycles. The mode of thawing and the temporary storage of thawed samples can also affect analytical readouts (95).

Lipid oxidation is usually not an issue for the quantification of abundant lipid classes (e.g., phospholipids, sphingolipids, and TGs), but could affect the analysis of lipid species that contain polyunsaturated fatty acid moieties, oxidized lipids, and eicosanoids, and may occur during collection, storage, and lipid extraction (56, 96). Addition of antioxidants immediately after obtaining plasma/serum samples, during storage, or before extraction may be required to limit ex vivo lipid oxidation (96, 97). Storage of plasma/serum samples and their extracts under an inert gas (e.g., argon) may also limit oxidation. However, ex vivo lipid oxidation may also occur enzymatically, which cannot be inhibited by the addition of antioxidants (56). Interestingly, in the case of butylhydroxytoluene (typically abbreviated as BHT), a frequently used antioxidant in lipidomics, the used concentrations and the time-point of its addition vary in the literature (98, 99). While it makes sense to take all reasonable precautions to ensure the chemical preservation of lipids, it seems sensible to first adhere to a minimalistic recipe. We therefore suggest that the efficacy and protocols for the use of antioxidants should be verified by the community for various lipid classes.

## ANALYTICS

Here, we define “analytics” as a collection of MS-based methods and software employed for identifying and quantifying plasma lipids. Although, from an analytical chemistry perspective, lipids are classical “small molecules,” the exact methods of small-molecule analytics are generally not applicable in lipidomics. Below, we briefly address the features of the most common sample preparation and analysis workflows that are immediately relevant for full-lipidome quantification.

### Analytical batches

An analytical batch is defined as a set of study samples that is processed and/or analyzed in a single continuous experimental setup (e.g., plate or day). The size of a batch is a tradeoff between sample throughput and technical/practical feasibility. A single batch should allow a consistent and reproducible analysis with minimized temporal effects. Differences in the experimental conditions (i.e., reagent lots, self-made mixes of ISTDs, pipettes, or instrument conditions) can lead to so-called batch effects, which are defined as systematic differences between batches of samples together with smaller differences between samples within each batch. However, “within*-*batch” variations can also occur, for example, intra-batch drift caused by temporal differences in sample processing (first vs. last sample), temperature shifts, and evaporation. These differences may or may not be correctable (see the Post-Analytics section).

To avoid batch-dependent biases and subsequent spurious correlations, stratified randomization of study samples is essential, considering key study factors across batches (i.e., treatment, age, or gender groups). Spatially stratified randomization may also be important when processing samples using multi-well plates and robotic systems. Information for each sample (preparation and analytical batch) should be reported even when batch corrections have been performed, because such information on batch-to-batch variation can help to improve analytical methods.

### ISTDs

ISTDs are critical elements to determine bona fide concentrations of lipid analytes. The use of ISTDs in MS also helps to compensate for inherent variations in sample processing (e.g., variations in lipid extraction efficacy, lipid class-dependent losses, matrix effects, and ionization suppression) and in instrument performance. ISTDs used for quantifying lipids should be added before lipid extraction. Ideally, an ISTD should be structurally similar and have a comparable MS/MS fragmentation pattern as the compound being quantified. In a method for 150 eicosanoids, 26 deuterated ISTDs were employed allowing excellent and reproducible quantification of plasma samples (100). In plasma lipidomics, we are committed to quantifying the molar abundance of hundreds of lipid molecules. However, using an authentic ISTD for quantifying each individual lipid is currently not possible or feasible. Therefore, the mass spectrometer, the analysis method (e.g., MS or MS/MS, precursor or neutral loss scanning) and the analysis conditions (e.g., analyte concentration range and buffer composition) should be selected such that a very limited set of ISTDs (typically, one to two molecules per lipid class) adequately reflects the quantitative properties of the measured molecules in the lipidome.

Recommendations concerning the number, concentration, type, and characteristics of ISTDs may be helpful toward harmonization of datasets. Defining a consensus minimal set of ISTDs or commercially available ISTD mixes for analyses may be useful. Different analytical approaches (e.g., direct infusion vs. LC-MS) may require distinct ISTD sets; however, common platform-independent ISTD mixtures may be advantageous. The ISTD concentrations should be close to the physiological values typical for correspondent lipid classes. Isotope-labeled analogs of endogenous lipids represent gold standards of ISTDs for plasma lipidomics, irrespective of the platform used. However, reliable ISTDs with adequate chemical purity and exactly known lipid content are only available for a limited number of lipid subclasses and fatty acid compositions. The use of several ISTDs per lipid class, with a wide range of acyl chain length and degree of saturation, may improve species quantification. Other strategies, such as using a combination of internal and external calibrations, may be more practical and affordable for large-scale analyses. A combined approach of using self-prepared (and therefore more affordable) stocks of ISTDs that are validated using high-quality commercial standards is another practical way to balance the costs (17).

Many plasma lipids are bound to soluble carrier proteins (e.g., albumin) or associate with multiprotein assemblies (lipoproteins) and extracellular vesicles. Some lipids may not be efficiently separated from their carriers and precipitate with proteins during extraction. The protocol and matrix in which ISTDs are added to the plasma samples (e.g., as a low-volume spike or as part of the extraction mixture) might result in differences in how much ISTD is co-extracted compared with endogenous counterparts. Preparing standards in surrogate matrices (e.g., stripped or native plasma), which is a widespread practice for clinical assays for exogenous compounds, is controversial in lipidomic applications. Surrogate matrices can be sources of contamination or increased background, or contain minute amounts of endogenous species.

Thus, each dataset should report information on ISTDs, including their chemical name, position of isotope labels, origin, and possibly also the lot number, concentration, chemical and isotopic purities, solvent/matrix, storage conditions, and when they were added to the samples.

Inconsistencies between individually prepared ISTD solutions (from stock or original ISTDs) are important sources of variation in quantification. Variability of concentrations of in-house-prepared standards is associated with differences in quality, chemical stability, and limited solubility of synthetic lipids, as well as preparation errors and evaporation of volatile solvents from stock solutions. Regardless of their precise origin, analytical inconsistencies could be recognized and appropriately corrected without jeopardizing the integrity of the entire project by implementing lipid standard validation protocols. The use of commercially available ready-made ISTD mixes with exactly known concentrations of individual lipids may nevertheless be the more robust and consistent approach in high-throughput clinical analyses.

The use and development of novel comprehensive and easily available isotope-labeled ISTD mixes should be further encouraged by the lipidomics community. An example of such ISTD mixes is commercially available ISTD mixes containing isotope-labeled species from all major phospholipid classes (4, 101).

### Lipid extraction

Lipid extraction from plasma or serum samples is the key step to eliminate the protein matrix and ensure the compatibility of samples with downstream analytical methods. However, lipid extraction is also a major source of variability between samples and between methods. The employed protocol should therefore be shared in detail and at least cover the major aspects described below.

There are numerous published lipid extraction methods that can also be automated for high-throughput clinical analysis (26, 71, 102–107). The applied extraction principles (e.g., one/two-phase liquid-liquid extraction, solid-phase extraction) and the parameters of extraction protocols (e.g., temperatures, sample/solvent ratio, re-extractions, use of sonication, vortexing, extraction under inert gasses, mixing and centrifugation details) are the major determinants of lipid recovery; however, they are also responsible for artifacts in lipid identification and inconsistencies in quantification. For instance, reconstitution in different volumes or different solvent may affect overall lipid recovery; whereas drying of lipid extracts in a heated vacuum concentrator may cause oxidation and biased loss of lipids due to binding to plastic or glass surfaces. The production of novel species ex vivo can also induce artifacts and affect recovery. Examples of such artifacts include enzymatic hydrolysis leading to the formation of lysophospholipids from the respective phospholipids, diacylglycerols from TGs, and free cholesterol from cholesteryl esters, as well as acid-induced chemical reactions, such as acyl and phosphate-migrations. We therefore encourage the lipidomics community to further compare, validate, and develop existing or novel methods for robust high-throughput compatible extractions of plasma samples.

It is unlikely that a single unified lipid recovery protocol could serve different analytical approaches: the diversity of lipid extraction and analysis protocols is unavoidable. However, on a positive note, the allowed flexibility in analytical routines is important for further method development, and one should not try to reduce it to some self-proclaimed “gold standard” methodologies. It is critically important to always include reference plasma samples in analyzed batches and report data as molar concentrations; this allows early identification of biases and systematic or occasional inconsistencies. Reporting lipid concentrations in a transparent format allows the data to be compared across independent studies and could spare the field from major interpretation biases.

### Quantitative lipid profiling by direct infusion, LC-MS, and LC-MS/MS

Plasma lipids can be analyzed by a range of MS-based methods, and currently no method dominates the field. LC-MS, direct flow injection (108), and direct-infusion/shotgun MS (DIMS) are the most common approaches yielding different data in terms of coverage, specificity, and sensitivity. However, within each approach, there is substantial variation in methods and software owing to the properties of the mass spectrometers and/or the type of chromatography.

The analysis of very low abundant (nanomoles per liter range) lipid mediators (e.g., eicosanoids, specialized pro-resolving mediators, oxysterols) is exclusively targeted and relies on LC-MS/MS in multiple reaction monitoring (MRM) mode (on triple quadrupole) (100, 109) or parallel reaction monitoring (PRM) mode (on quadrupole TOF or hybrid quadrupole Orbitrap) (52), or high-resolution selected ion monitoring (on Orbitrap) MS. More abundant lipid classes in the high micromoles per liter to millimoles per liter range (e.g., glycerolipids, glycerophospholipids, cholesterol esters, and ceramides) can be analyzed by DIMS as well as by LC-MS and LC-MS/MS. DIMS platforms offer robust high-throughput analysis of large sample sets due to relatively short run times, simple set-up, and easy maintenance (99, 110). Importantly, lipid analytes are ionized together with ISTDs and the analyte composition does not change with time; this equalizes matrix effects, although at a cost of considerable and matrix-dependent ion suppression, and potentially simplifies quantification. On the other hand, DIMS analyses result in highly convoluted spectra and, due to matrix interference, lipid class coverage and sensitivity toward individual components are generally lower compared with LC-MS methods. Matrix interference can be a significant source of variability in any MS analysis and may thus impact quantification when only one or a few ISTDs are used; this is especially the case in LC-MS, where lipid species are separated over the chromatographic space.

Therefore, a truly comprehensive lipidome analysis might require the parallel use of several analytical platforms (each suited for a subset of lipid classes), the application of specific ISTDs, and particular sample preparation techniques. Frequently, plasma lipidome analysis is focused on a selection (typically 20 to 25) of the most abundant lipid classes that can be analyzed in a single run on the available mass spectrometer.

In summary, the palette of analytical approaches reflects the diversity of physicochemical and structural properties of individual constituents of the lipidome. It is also guided by the research goals and priorities of throughput versus molecular specificity. Diverse analytical platforms are bound to coexist, and the lipidomics community faces the challenge of creating a framework that harmonizes and cross-validates the data acquired across different projects. A generally adopted requirement to report absolute (molar) concentrations of analyzed plasma lipids, irrespective of employed methodology and study design, could be a first step toward this goal.

### Experimental QC samples

In addition to ISTDs, reference and QC samples should be processed and analyzed with study samples within each experimental batch. These should address process and instrument variations to track analytical fidelity of the experimental workflow (27). Examples of these are: *i*) Batch control samples to monitor and potentially compensate for variation between individual batches; these batch QC (BQC) samples could include plasma samples of the same defined source (e.g., preferentially a pool of a representative subset of the study samples) that are sequentially and intermittently processed with the study samples using the exact same procedures applied to the samples. The study and BQC samples are then analyzed in the same sequence as they were processed. *ii*) Invariant matrix control samples are also required to assess the stability of the analytical platforms, i.e., a technical QC (TQC) sample, which is a repeatedly injected control sample (e.g., pooled BQC extracts or other reference samples) that can be used to monitor equilibration and performance of the LC-MS instrument over time. Ideally, a TQC is the most invariant QC sample over time for a given matrix. *iii*) Control samples are also required to determine the analytical performance toward individual lipids, i.e., coefficient of variation (CV), blank-sample ratio, limit of detection and lower limit of quantification, linearity, and stability.

Blanks should be prepared using the same containers, solvents, and procedures as the study samples, by extracting the same ISTD mix in the absence of plasma or serum. Extracted blanks should also be analyzed at regular intervals throughout the batch.

The ratio of QC samples to study samples depends on sample size, analytical requirements, and experimental setup. Guidelines such as the FDA and EMA Bioanalytical Guidelines (35, 36) detail the use of specific QC samples. QC samples for testing lower and upper limits of detection are usually not applicable for lipidomics analyses, but might be used in panels for specific lipids that have corresponding isotope-labeled ISTDs. In general, it is advisable to have QC at low clinical decision points and high concentrations. Signal saturation and dilution-related effects can occur in direct-infusion and also in LC-MS analyses. Dilution series of QC (i.e., TQC) samples can provide information for each measured lipid species on linearity of the responses.

### Standard reference materials

The NIST SRM 1950 plasma is a well-accepted and characterized standard reference plasma for which comprehensive lipidomics analyses were conducted (12, 13, 74). It was prepared in 2007 from 100 donors representing the ethnic distribution of the US and with a female to male ratio of 1:1. All donors were “healthy,” as judged by a limited health check. Biases in specific lipids caused by conditions present in a few donors might be possible. Absolute concentrations of diverse metabolites, including specific lipids such as fatty acids and steroids, estimated with independent approaches, are available (74, 111). As noted above, the NIST SRM 1950 plasma has been collected from lithium heparin anticoagulated blood, whereas EDTA is the predominant anticoagulant in lipidomics research (74).

Recently, the NIST SRM 1950 plasma was analyzed by a study group comprising 31 laboratories, which used various quantitative analytical methods, relying on LC-MS/MS and direct infusion (shotgun) profiling on different mass spectrometers (13). The study group reported consensus values of absolute (molar) concentrations of specific species from different lipid classes that rely on the concordance between independent measurements. These data now can be used in comparisons in future work.

Using the NIST SRM 1950 as a reference plasma will not only be useful in harmonizing datasets but will also provide valuable information on the analytical variability across approaches, platforms, and software, recognizing problematic lipid species and classes whose quantification is “consistently inconsistent” between sites, identifying platform-dependent quantification biases, and, hence, enabling the continuous improvement and standardization of quantitative plasma lipidomics. The routine use of SRM for research is not trivial, given the limited quantity and high cost of using such material. However, this is the only way to reach quantification consistency across the entire lipidomics community and, eventually, to integrate lipidomics in clinical chemistry routines world-wide. To reduce the costs and extend the life of the NIST SRM 1950 repository, it might be useful to prepare and assign calibrator values to in-house reference materials (e.g., pooled plasma from other sources). Periodic comparison of in-house calibrators with SRM would then be a sensible next step toward standardization. The plasma lipidomics and metabolomics communities should also aim to estimate the future need, available supply, and quality of the NIST SRM 1950 plasma, and start planning for potential commonly acceptable alternatives for the future.

In conclusion, we strongly recommend analyzing and reporting the NIST SRM 1950 reference (for the moment) and employing QC samples so that datasets can be compared.

## POST-ANALYTICS

### Raw data processing

Processing of MS raw data to extract abundances of lipid species is another key aspect of lipidomic analyses. Data processing has a major impact on the reported data and data quality in untargeted data-dependent acquisition (DDA) and data-independent acquisition (DIA), as well as in targeted approaches. The default parameters of software tools are subject to change, and thus, all (not just the modified) parameters should be reported. When web services are used, the software version number and the date accessed should be documented. Methods applied to ensure correct peak picking and integration are also important considerations, particularly for targeted strategies (e.g., MRM). Criteria for manual input and data curation (e.g., manual peak integration) should also be documented.

We encourage researchers to provide detailed information in their data analysis workflow to help other researchers to reproduce data analyses and to optimize their own workflows, enable improvements in algorithms, and provide benchmarking of data processing tools. This would be akin to harmonization efforts in other fields, such as in proteomics with the “minimum information about a proteomics experiment (MIAPE)” guidelines (112). For plasma lipidomics, our starting situation is particularly opportune, especially in combination with: *i*) standardized reference materials (described above); and *ii*) commonly agreed reporting of molar values. It is also timely, with several harmonization efforts underway concurrently, such as the Lipidomics Standards Initiative (see also the Immediate Outreach section below).

### Isotope interferences and response factors

Isotopic interferences can lead to an incorrect assignment of signals and errors in quantification, which can be caused by up to M+4 isotopologues. Particularly in DIMS and hydrophilic interaction chromatography (HILIC)-MS, isotopic interferences can affect lipid quantification. Longer chromatographic runs may allow better separation of species that might otherwise overlap, and thus they can reduce isotopic interferences. Post-analysis corrections can help to improve the quantification of the affected species, and changes in the relative isotope distribution, depending on the chain length, should be integrated into the calculations (113, 114). Furthermore, the fatty acid composition in complex lipids can considerably (several fold) affect their MS response and should be estimated and considered during quantification, using multiple class-wise ISTDs. Such corrections, if applied or omitted, can lead to significantly diverging concentration values, such as noted for cholesteryl esters (115).

### Lipid annotations

Plasma lipids are (and in the foreseeable future, will be) analyzed by diverse methods, each of which delivers different levels of structural specificity with respect to the identification of structurally unique molecules. For example, lipid identification relying solely on matching of accurately determined masses of intact molecules (i.e., as in the top-down shotgun lipidomics) will not distinguish lipids that belong to the same class and share the same number of carbon atoms and double bonds in their fatty acid or fatty alcohol moieties. If lipid identification also uses MS/MS and/or retention time matching, it becomes possible to distinguish molecules with unique fatty acid and fatty alcohol moieties. The exact positioning of double bonds could be further determined using ion mobility MS or ozonolysis (116).

To make datasets comparable, reported lipid names must be categorized, standardized, and drawn correctly depending on the level of identification (117–119). We strongly recommend the use of respective hierarchical nomenclature, such as PC 36:3 or PC 18:1_18:2, or further with positional information (119, 120). This distinction will help to match molar concentrations of plasma lipid species irrespective of analytical methods. For example, concentrations of isobaric molecular species of lipids quantified by LC-MS/MS could be summed up and compared with the total concentration of their entire pool determined by top-down shotgun lipidomics. Similarly, it should be possible to compare the summed concentration of several lipid classes with integral indexes determined by clinical blood tests (17). In this comparison, the total cholesterol index would reflect the sum of concentrations of free cholesterol and cholesterol esters. Similarly, total TG index reflects the summed concentration of all measured glycerolipids.

### Data QC

Processed datasets should be subjected to a rigorous QC process to check and potentially remove artifacts at the species and dataset levels. A first line of data QC procedures should aim to filter lipid species that do not fulfill specific criteria, e.g., as defined in the FDA/EMMA guidelines for bioanalytical methods (35, 36). CVs for analytes in the QC samples (preferably BQC samples, see above) should generally be within 20%, a common threshold in the literature. However, FDA and EMA recommend a maximum CV of 15%, except at the lower limit of quantification (35, 36). Dilution series of a QC (i.e., of TQC) sample provides information for each measured lipid species in terms of the linearity of response: only signals with a linear response should be considered for quantification. Reported species should have been quantified in at least 95–99% of samples to avoid issues with missing values and to avoid reporting species that cannot be reliably quantified. Lipids that were monitored in a targeted analysis, but not detected, should also be reported and clearly indicated as “not detectable.”

Specific metabolites and lipids, such as sphingadienine 1-phosphate (S1P d18:2), have been proposed as markers for plasma and serum quality and preanalytical conditions (77, 121–124). Although these markers still have to be validated in larger cohorts and in patients with different diseases and under different treatments, the measurement of several independent markers of sample quality would add valuable information to the lipidomics datasets, enabling better interpretation of data and the identification of potential artifacts, especially when analyzing samples from existing biobanks.

Harmonized and commonly applied minimal sets of QC procedures and criteria will improve data quality. We encourage databases and journals to require a certain set of QC information, i.e., CVs and results from a reference material (such as the NIST SRM 1950) for data submission.

### Quantification

Quantification of measured lipids in standardized concentration units is essential for comparison and interpretation of shared data and a prerequisite for clinical research applications. Reported concentrations should be expressed in the SI unit, moles per liter, whenever possible, depending on the analytical approach. Ultimately, this will allow for a true comparison. The percentage of total lipids or molar percentages depend on the applied method and coverage. Molar percentages are informative and legitimate; yet we would like to encourage reporting of molar concentrations whenever possible, which would still allow subsequent calculations of molar percentages.

Calculation of individual lipid concentrations from lipidomic datasets comprises different steps and requires specific assumptions that will affect estimations: *i*) normalization with the corresponding ISTD; *ii*) correction for isotopic overlap and isotope distribution effects; *iii*) normalization for the starting sample amount; *iv*) calculation of absolute concentrations based on spiked ISTDs, calibration curves, and response/correction factors; and *v*) drift/batch corrections.

#### Step i.

ISTDs can be used for the absolute quantification of lipids. The gold standard would be the use of stable isotope-labeled ISTDs for each measured lipid species. However, only a very limited number of such standards are available; thus the challenge is to perform the analysis such that concentrations of other lipids could be inferred from the abundances of ISTDs. This, in turn, is bound to rely on several nonobvious assumptions and the entire workflow needs to be independently validated.

#### Step ii.

Isotopic interferences can be numerically corrected by subtracting the theoretical isotopologue abundance from an affected species. However, such numerical methods have limitations and can decrease precision or be impossible altogether, e.g., when the contribution of the interference is considerably higher than the actual signal from the analyte. Also, isotopic correction algorithms are method dependent. Different algorithms are applied for MS- and MS/MS-based quantification and should be adjusted to mass resolution of the employed instrument (113, 125). Applied isotopic correction methods and corrected species should be documented, which will help to identify potentially problematic corrections.

#### Step iii.

For plasma and serum samples, normalization with the sample volume is the single most robust and accepted strategy for reporting absolute concentrations. The blood and plasma volumes are highly regulated and the within-subject biological CV of the hematocrit is approximately 3% (126). The use of other signal normalization approaches, such as total ion counts, the sum of measured lipid abundances per class, or the total and protein levels, are prone to artifacts, as they depend on the analytical method as well as the sample. This is of special concern, as clinical samples might have different protein, total metabolite, or lipid levels. Thus, we discourage the use of such alternative methods for normalization and quantification.

#### Step iv.

Calibration curves for ISTDs and assay-specific response factors for particular lipids in standard addition experiments are required for absolute quantification to compensate for species-specific, concentration-specific, and matrix-dependent effects, given the defined ISTDs. Plasma samples can have considerable variations in matrix effects, especially in workflow relying on separation of lipids by reversed-phase chromatography (127); thus, the robustness of the response factors in different plasma samples should be established in both healthy subjects and patients.

#### Step v.

Systematic variations within and between analytical batches may be present even after normalization with ISTDs in the form of continuous and discrete shifts. Drift and batch effect correction methods modify data and may themselves introduce errors and variation (e.g., due to model overfitting). When “global” correction methods are applied to a dataset, individual lipid species might not be appropriately corrected. We therefore recommend performing these corrections at a lipid species level, as lipids from different classes and chain lengths or saturations may be differently affected by drift/batch effects. The application of correction methods should be done conservatively and only when there is statistical evidence for batch or drift effects. Models, parameters, and the magnitude of performed corrections should be reported.

Above, we have pointed to a few of the most common (and, hence, well-understood) issues in lipid quantification. However, practical implementation of quantification routines is dependent on the instrument platform: for example, isotopic correction algorithms heavily depend on the mass resolution and are different for low-resolution (triple quadrupole), high-resolution (TOF), and ultra-high-resolution (Fourier-transform ion cyclotron resonance or Orbitrap) instruments. Considering the method differences, the community should not enforce strict guidelines for the method of lipid quantification, such as those applied to the quantification of pharmaceuticals and their metabolites. Therefore, we underscore the value of absolute (molar) quantification and encourage researchers to provide data for cross-platform comparisons, which could identify unavoidable platform-specific biases. Openness and transparency of reported datasets will work more efficiently than the most comprehensive and stringent guidelines.

### Data sharing

Depositing raw data (original unprocessed data generated by the instruments, e.g., spectra, MRM chromatograms) serves as experimental proof and allows other researchers to independently reanalyze data to test different hypotheses or to validate findings. However, such reanalyses still rarely appear in the scientific literature, which may be explained by lack of dataset transparency, poor organization of the associated meta-data, and difficulties in processing vendor-specific file formats. Submission of absolute concentrations of lipids in each analyzed sample combined with the responsibility to provide associated raw data would be a practical compromise. Nevertheless, deposition of raw data may allow for the systematic and automated reanalysis of experiments with improved or novel computational approaches. Centralized and uniform processing of raw data from different experiments across platform sites and samples is important to achieve a much-needed harmonization of datasets. An open format, like mzML, that is compatible with MRM, PRM, DDA, and DIA data is preferred for raw data submission (128). Efforts to obtain better data conversion tools and support from vendors for the transfer of proprietary raw data formats to open formats should be intensified and become a part of the instrument acquisition process (129). Open formats of MS raw data may be essential in the future for clinical applications and would enable the community to develop better data analysis algorithms in the field of lipidomics.

Reporting analytical details and results should be done primarily in consistent “machine-readable” formats based on XML or structured tabular formats. The proteomics community has established several open formats for data exchange from proteomics experiments, between software types, and for data sharing (130, 131). These file formats include mzQuantML and mzTab. TraML is a file format for the exchange of targeted MS/MS transition lists, and the qcML format provides QC information of the instrument used for the analysis of samples, e.g., mass accuracy. Recently, efforts are being made to further adapt and define these formats for metabolomic and lipidomic applications (132). Reported data should also include all relevant QC data, such as the CVs of the measured lipid species and data from reference materials, among others. For reporting of diagnostic/analytical performance in clinical studies, an alignment with the Standards for Reporting Diagnostic Accuracy (STARD) standard should be considered (133).

Journals publishing lipidomics data should also enforce reporting all the experimental, analytical, and data processing details. *Molecular and Cellular Proteomics*, for example, has recently updated their data disclosure and deposition requirements (see http://www.mcponline.org/content/data-reporting-requirements and http://www.mcponline.org/page/content/clinical). Some journals, including *Nature Scientific Data*, require the full submission of data and associated information using ISA-Tab data format files (http://www.isacommons.org) (134). The lipidomics community could consider negotiations with relevant journals in defining minimal sets of data to be reported for the submission of lipidomics data. The lipidomics community should furthermore actively participate in the current efforts to integrate and enhance metabolomics/small molecule information into established open XML-based MS data formats, to define novel reporting formats for targeted method parameter, QC data, clinical parameters, and other information of relevance to clinical “omics” research.

Plasma lipidomic applications are a well-defined and relevant starting point for efforts toward defining the reporting and data exchange formats of clinical omics data and associated information. The focus of this position paper is on quantitative plasma lipidomics. Although various databases/repositories for metabolomics and lipidomics data exist (e.g., Human Metabolome Database, MetaboLights, MassBank, LIPID MAPS), they are mostly focused on raw data and spectral information (118, 135–138). Human Metabolome Database entries can contain information on metabolite concentrations in biological samples; however, any associated data is in the form of free text and links to references. What is currently lacking and is urgently needed is a repository that allows systematic storage and structuring of lipid concentration data, with associated analytical and clinical data. Recently, LIPID MAPS, in collaboration with the Metabolomics Workbench, has implemented an open data repository for large lipidomic datasets (https://www.lipidmaps.org).

### Regulatory aspects of data sharing

Country-specific regulations restrict the storage and sharing of human data in central repositories. Omics datasets may allow for the re-identification of subjects from de-identified datasets based on omics profiles and the reported demographic and clinical data. Strategies should be developed at different levels to enable the best use of datasets for research, while warranting data privacy and compliance to national and international laws and regulations. These strategies may include: *i*) including the potential international sharing of samples and/or data derived from the samples in ethics approval and patient consent forms; *ii*) using sampling protocols that allow for the proper de-identification (e.g., large enough participant pools to allow for full anonymization or de-identification); *iii*) proper and internationally accepted de-identification procedures (e.g., the researcher involved in sample analysis must not be in a position to re-identify samples); and *iv*) using data structures and information technology infrastructures that enforce and ensure data privacy and security.

## IMMEDIATE OUTREACH

This work has been inspired by the widespread understanding of the importance for standardized analytical protocols, methods of spectra processing, lipid quantification, and the reporting of lipidomics data. In the field of molecular medicine, lipidomics is currently recognized as a discovery tool. However, it is rapidly expanding into neighboring areas, such as clinical research and personalized monitoring. It is conceivable that, in the future, plasma lipidomics will draw attention of practicing physicians, similar to many established clinical chemistry indexes. That said, reporting lipidomic data should eventually adhere to common clinical format and we anticipate that this work could serve as the first step in this direction.

Plasma lipidomics relies on diverse analytical platforms, and inter-laboratory concordance of lipids quantification is not ideal. Yet, we argue that the first step toward harmonizing plasma lipidomic data produced in different laboratories could be the commitment to report molar concentrations of individual lipids. This, however, does not alleviate the need to include clinically relevant meta-data and describe analytical and statistical procedures as well as to deposit raw data on dedicated resources in the public domain.

It is difficult to project how quickly this initiative will progress. Other initiatives aimed at standardization of essential steps in the lipidomic characterization of biological specimens are underway (e.g., Lipidomics Standards Initiative, https://lipidomics-standards-initiative.org). These are well-aligned with the specific example of plasma lipidomics outlined here. Efforts toward promoting the exchange of data and methods, open discussions on the methodological and clinical issues, and increased awareness of granting agencies and journals will be of significant help. Let us remind ourselves that common clinical indices reported by a clinical blood test and now unequivocally interpreted by any qualified physician world-wide were not established instantly, but emerged during the lengthy and laborious process of harmonizing operation procedures and output formats. The lipidomics community should follow the same path.
